# Expression and purification of a gene encoding a 9.7 kDa PE protein of *Mycobacterium avium* subsp. *paratuberculosis*

**DOI:** 10.1007/s13205-016-0506-7

**Published:** 2016-09-15

**Authors:** S. Chandra Sekar, P. P. Goswami, R. Deb

**Affiliations:** Division of Veterinary Biotechnology, ICAR-Indian Veterinary Research Institute, Izatnagar, 243122 Uttar Pradesh India

**Keywords:** Map, PE, pQE30 UA, Purification, DTH

## Abstract

*Mycobacterium avium* subsp. *paratuberculosis* (Map) contains PE family antigens which are Proline and glutamic acid rich and may play important role as T-cell antigens. In the present study, the Map 1507 ORF encoding 9.7 kDa PE protein was amplified by polymerase chain reaction and cloned into *E. coli* vector pQE30 UA. The recombinant plasmid designated as pQ PE was transformed into *E. coli* M15 cells and induced with IPTG revealed the high level expression of 11.9 kDa His-fusion protein as estimated by migration in 15 % sodium dodecyl sulfate polyacrylamide gel electrophoresis (SDS-PAGE). Recombinant PE protein was purified by Ni–NTA agarose chromatography. Polyclonal antibodies raised against purified recombinant PE protein reacted with expressed PE protein as well as with Map sonicate. The recombinant PE protein was also recognized by serum from goat with clinical paratuberculosis. The protein elicited significant delayed type hypersensitivity (DTH) skin reaction in mice sensitized with Map. The results indicated that the recombinant PE protein of Map was associated with T-cell response.

## Introduction

The search for *M.a.paratuberculosis* (Map)-specific antigens for diagnostic or preventive therapy has led to the discovery of several immunoreactive proteins. Many of these proteins have homology to other mycobacterial antigens.

Little is known about the structure, function, or immunological response to the PE proteins encoded by the subfamily of PE genes found throughout the genome of *M. tuberculosis* and other mycobacteria (Khubaib et al. 2016; Deng et al. 2015; Fishbein et al. 2015; Brennan et al. 2005; Fleischmann et al. 2002). These PE genes encode proteins that range in size from ~30 to ~110 amino acids, and most contain a characteristic Pro-Glu (PE) amino acid motif near the N terminus. Similar sequences are found as the N-terminal domain of the larger subfamily of proteins that contain polymorphic glycine repeat sequences (PE_PGRS) (Brennan and Delogu 2002). Studies using reverse transcriptase PCR and microarray analyses (Fisher et al. 2002; Voskuil et al. 2004) revealed that certain PE genes are expressed by *M. tuberculosis*. It has also been reported that PE 35 gene that was found in RD1, a multigene region that is absent in Mycobacterium bovis BCG strains, can elicit an immune response (Brusasca et al. 2001). *Mycobacterium avium* genome contains only a few PE genes and no PE_PGRS genes (Parra et al. 2006).

Parra et al. (2006) have shown that a PE protein of *M. avium* is a potent T cell inducer and capable of eliciting significant amounts of IFNγ in experimental mice model. Immunization of mice with a novel PE gene expressed by *M. avium* (MaPE) showed that a dominant T-cell immune response was elicited. Immunization with a MaPE DNA vaccine protected mice against an aerosol challenge with *Mycobacterium tuberculosis*, suggesting that mycobacteria expressed PE antigens with cross-protective T-cell epitopes. Hence the identification and expression of PE antigen that stimulate T cell response seem to be an initial requirement for the development of effective vaccines and or CMI based diagnostic. The present communication deals with the cloning expression, purification and preliminary characterization of the 9.7 kDa PE protein of Map in *E. coli*.

## Materials and methods

### Mycobacterial strains

Bacterial strain of Map 316F were obtained from Central Diengenees Kunding Tieh Instituut, Lelystad, The Netherlands in Biological Products Division of IVRI, Izatnagar, and later maintained at Gene Expression lab, Division of Animal Biotechnology. IVRI, Izatnagar.

### Plasmid and host strains

pTZ57R/T Cloning vector and host strain DH5α of *E. coli* were supplied by MBI Fermentas, Germany. Prokaryotic expression vector pQE30UA was purchased from QIAGEN, Germany.

### Laboratory animals

Swiss albino mice and New Zealand white rabbits were obtained from Laboratory Animal Resource Section, IVRI, Izatnagar. Standard prescribed guidelines for care and use of laboratory animals were followed during the experimentation with these animals.

### Culture and growth of Map and *E. coli*

Map organisms were grown on Middlebrook 7H10 agar enriched with 0.1 % glycerol v/v and 10 % oleic acid dextrose catalase (OADC) with additional supplementation of Mycobactin J (2 mg/l) and were maintained at 37 °C. *E. coli* cells were grown in Luria–Bertani (LB) medium at 37 °C with shaking at 180 rpm. For the preparation of LB plates, 1.5 % agar powder was added to LB medium prior to autoclaving. Appropriate antibiotics were included as per requirements.

### Isolation of genomic DNA form Map

The genomic DNA from Map was isolated by the method of Portillo et al. (1991) with a few modifications. The bacterial colonies were scrapped from 2-month-old Middle brook 7H10 agar slants in 1.5 ml microfuge tube, washed thrice with 1X TE and resuspended in 500 µl of 1X TE. Lysozyme was added to the final concentration of 5 mg/ml and incubated at 37 °C for 2 h. SDS and proteinase K were added to a final concentration of 1 % and 250 µg/ml, respectively, and incubated further at 65 °C for 30 min. To this, 80 µl of 5 M NaCl was added and vortexed. This was followed by addition of 64 µl of CTAB/NaCl solution and vortexed. The suspension was again incubated at 65 °C for 30 min. DNA was extracted once with phenol, once with phenol: chloroform (1:1) and finally with chloroform: isoamyl alcohol (24:1). The aqueous phase containing DNA was pelleted by centrifugation and washed with 80 % ethanol, dried and redissolved in 200 µl of 1X TE. Contaminating RNA was removed from DNA by incubating with 100 µg/ml RNase. The treatment was given for 1 h at 37 °C, followed by phenol:chloroform extraction and ethanol precipitation.

### Cloning of PE gene in a prokaryotic expression vector pQE30UA

Specific amplification of the PE gene (Forward: 5′-GCC GCT AGC ATG TCG TTC GTG ACC ACA CA-3′ and Reverse: 5′-GCC GAA TTC TCA GAG GGC CGC GGC GGC GT-3′) from the genomic DNA of Map was carried in a 25 μl reaction volume containing 1 μl of genomic DNA (10 ng) as template, 2.5 μl of PCR buffer, μl of MgCl_2_ (1.5 mM), 1 μl (25 μM) of each primers, 1 μl of dNTP mix (200 μM of each dNTP) and 1 U of Taq DNA polymerase. The volume was made up to 25 μl by adding DNase free water. The thermal cycling steps were carried out in PTC-200 thermocycler MJ Research Inc, USA with initial denaturation at 94 °C for 5 min followed by 30 cycles with denaturation at 94 °C for 1 min, annealing at 53.8 °C for 1 min, extension at 72 °C for 30 s and final extension at 72 °C for 10 min. Size of the amplified product was confirmed by using DNA molecular weight marker in a 1.2 % agarose gel and quantified by spectrophotometric analysis. The PCR amplified gene product was purified from agarose gel using a QIAEXII gel extraction kit (Qiagen, USA) and ligated to pQE30 UA expression vector the resulting plasmid designated as pQEPE. On transformation into *E. coli* M15 host cells the recombinant clones were selected on LB agar containing ampicillin (100 µg/ml) and kanamycin (25 µg/ml). Transformants were further screened by colony PCR, restriction enzyme analysis of the plasmids with *Nhe*I and *Eco*RI. Positive clones were send for sequencing. The nucleotide sequence of the 300 bp gene fragment encoding 9.7 kDa PE protein from Map starin 316F has been deposited in nucleotide database. The deduced amino acid sequence of the 9.7 kDa PE protein was analyzed for hydrophobic domains according to Kyte and Doolittle algorithm (Kyte and Doolittle 1982) using Lasergene software (DNASTAR, Madison, USA).

### Expression and purification of the recombinant PE protein

Overnight grown cultures of the selected recombinant were inoculated in LB broth containing (75 μg/ml) and kanamycin (25 μg/ml). Once an optical density at 600 nm (OD 600) of the culture reached 0.6–0.7, cells were induced with 1.0 mM isopropyl thiogalactoside (IPTG) for 4–6 h. Whole-cell lysate of the bacterial were prepared and the expression of the recombinant PE protein from the induced and uninduced culture and *M15* cells were collected, lysed in sample buffer and stored at −20 °C for SDS-PAGE. The purification of the recombinant PE protein (9.7 kDa PE and 2.2 kDa N terminal fusion domain of pQE30UA) was purified by Ni–NTA (nickel-nitrilotriacetate) agarose affinity chromatography (Qiagen) After induction, cells (0.5 g) was lysed by stirring in lysis buffer (6 M GuHCl; 0.1 MNaH_2_PO4; 0.01 M Tris–Cl; pH 8.0) for 30 min, centrifuged at 10,000*g* for 20 min. Lysate was again mixed with 50 % Ni–NTA slurry (4:1) and kept at 4 °C for 1 h. Ni–NTA slurry was loaded on the column and finally the purified protein was eluted using elution buffer (8 M Urea; 0.1 M NaH_2_PO_4_; 0.01 M Tris–Cl; pH 4.5) after washing with wash buffer (8 M Urea; 0.1 M NaH_2_PO_4_; 0.01 M Tris–Cl; pH 6.3 and pH 5.9). The fractions containing recombinant PE protein were extensively dialysed at 4 °C against PBS, to renature the protein. The protein concentration was determined spectrophotometrically. The protein solution was sterilized by filtration and aliquots were stored at −70 °C, until used.

### Hyper immunization for raising antisera against Map PE antigen

Swiss albino, 6–8 weeks old mice, (six mice) were injected subcutaneously with 100 μg of purified recombinant PE protein emulsified in incomplete Freund’s adjuvant (IFA) (Genei, India). After 2 weeks subsequent two boosters were given at 2 weeks intervals. The mice were bled a week after the last dose and serum was collected later and stored at −20 °C in aliquots for western blot analysis.

### Sero reactivity of PE antigen

Expressed recombinant PE protein as well as Map sonicate were separated by 15 % SDS-PAGE under reducing conditions and then electro blotted onto a nitrocellulose membrane (0.45 μm) in a transfer buffer containing 48 mM Tris–HCl, 39 mM glycine, 0.037 % SDS, and 20 % methanol at pH 8.3, using a Semi-Dry Transfer System (Atto, Tokyo, Japan) at 0.8 mA/cm^2^, following the method of Bjerrum and Schaffer-Nielsen (1986). The blots were blocked with 2 % skimmed milk powder in PBS-T buffer (PBS containing 0.1 % Tween 20) for 2 h at room temperature. Immuno detection was carried out using polyclonal serum (1:2000, in PBS) raised in mice against recombinant PE protein followed by 1:500 dilution HRP-labeled rabbit anti-mouse IgG (Genei, India). Antigen was visualized on the blots by incubation with 0.02 % diaminobenzidine (DAB) suspended in PBS containing 0.03 % hydrogen peroxide. Further, about 25 μg of recombinant PE protein in 25 μl PBS and Map culture sonicate were put on nitrocellulose membrane dried and allowed to react with goat serum was obtained from a goat with clinical paratuberculosis as per the method described in western blot.

### Delayed hypersensitivity testing

Thirty-six female Swiss albino mice (6–8 weeks old) into two groups consisting of eighteen animals each, the group I was subcutaneously injected with recombinant PE protein (100 μg/animal in PBS) mixed with sterile incomplete Freund’s adjuvant (IFA) (1:1), while the group II (control) was immunized with PBS-IFA alone. Two weeks after the second immunization, mice were injected, with a tuberculin syringe into the footpads, with 10 μg of the recombinant PE protein or PPD (Purified Protein Derivative) in 0.02 ml of PBS. Six mice each from both groups were also injected with 10 µl PBS as negative control. The results of the local skin reactions (DTH) were read after 48 h by measuring the two transverse diameters of erythema and swelling using Vernier calliper. Differences between swelling observed before and after protein injected in footpad and the swelling observed in PBS injected footpad were noted.

### Statistical analysis

Data presented here are designated as mean ± Standard error mean. One-way ANOVA was employed to evaluate the statistical differences among groups with SPSS 13.0 software (SPSS Inc., Chicago, IL, USA). A value of *P* < 0.05 was considered as significant.

## Results

### Heterologous expression of PE antigen of Map

**Fig. 1:**
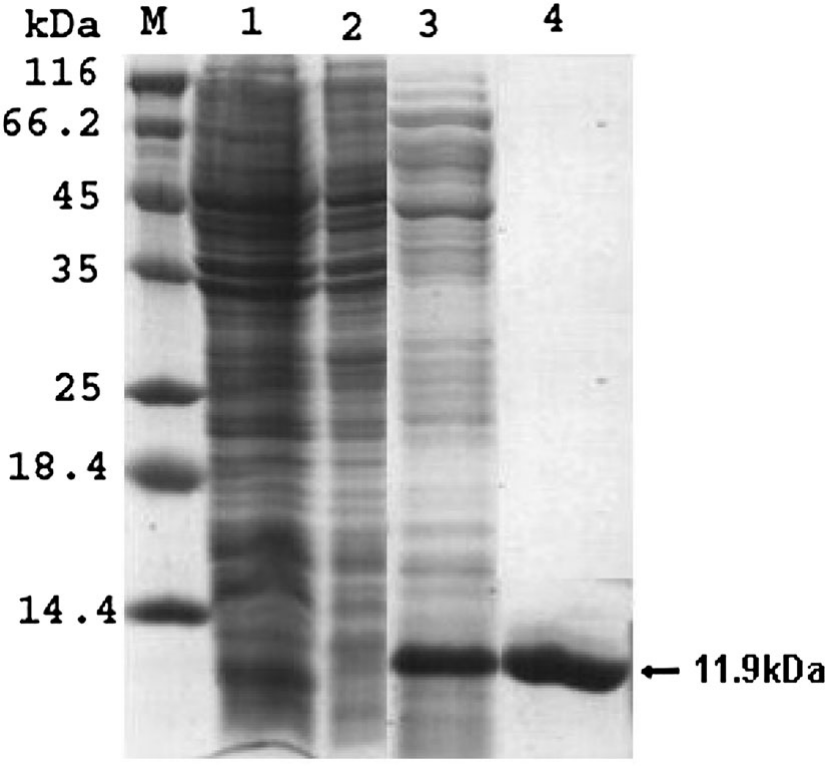
Coomassie Brilliant Blue stained 15 % SDS-PAGE gel, showing expression of recombinant PE protein. *lane M* prestained protein molecular weight marker, *lane 1* whole cell extract of *E. coli* M15 cells; *lane 2* whole cell extract of *E. coli* M15 cells harboring plasmid pQE PE (uninduced), *lane 3* whole cell extract of *E. coli* M15 cells harboring plasmid pQE PE (IPTG induced—6 h post induction); *lane 4* purified recombinant fusion protein His-PE

The PCR primers used in this study amplified a 318 bp fragment (300 bp PE gene and 18 bp linker), which was cloned in pQE30UA vector and the resulting plasmid pQEPE on restriction digestion with, *Nhe*I and *Eco*RI released an identical size fragment on 1.2 % agarose gel. The *E. coli M15* cells harboring the plasmid pQEPE on induction for 6 h with 1 mM/L IPTG resulting accumulation of insoluble 11.0.2 kDa His-fusion protein detected in total cell extract of *E. coli*., which is corresponding to that predicted for recombinant PE protein (Fig. 1, lane 3). No such protein band was observed with *E. coli M15* cells or in uninduced *E. coli M15* cells harboring recombinant plasmid pQEPE (Fig. 1, lanes 1, 2).

### Purification of the recombinant PE protein

The molecular mass of the recombinant PE protein obtained was estimated to be 11.9 kDa by 15 % SDS-PAGE (Fig. 1, lane 4). The yield of pure recombinant PE protein was about 18–20 mg/l of culture.

### Immunoreactivity of the recombinant PE protein

**Fig. 2:**
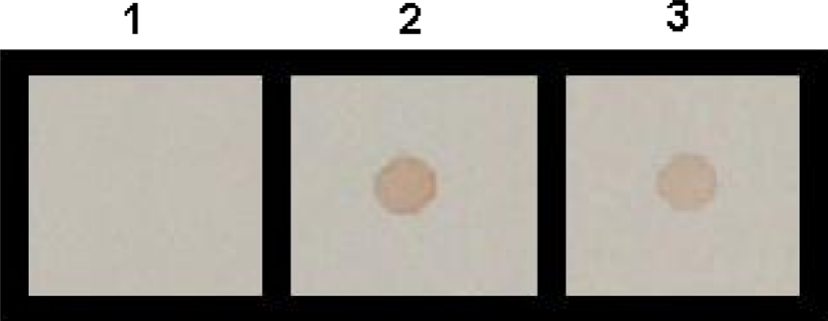
Dot blot assay of the recombinant PE protein. *1* Recombinant PE protein probed with control sera; *2* recombinant PE protein probed with hyperimmune sera; *3* *M.a.paratuberculosis* culture sonicate probed with hyperimmune sera

**Fig. 3:**
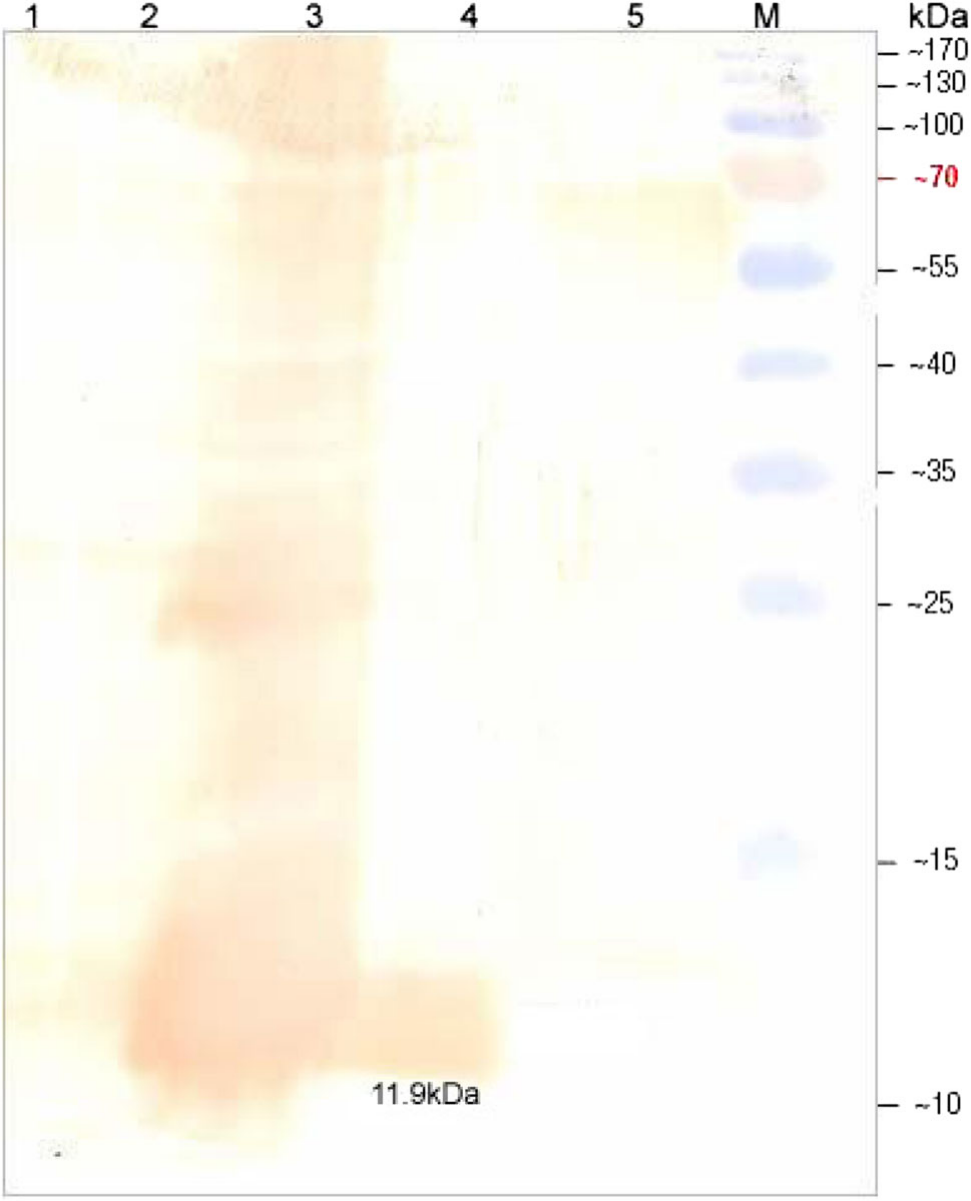
Western blot assay of the recombinant PE protein. *lane 1* whole cell extract of *E. coli* M15 cells; *lane 2* whole cell extract of *E. coli* M15 cells harboring plasmid pQE PE (uninduced); *lane 3* whole cell extract of *E. coli* M15 cells harboring plasmid pQE PE (IPTG induced—6 h post induction); *lane 4* purified recombinant His-PE protein; *lane 5* sonicated antigen of *M. a. Paratuberculosis*; *lane M* prestained protein molecular weight marker

Western blot analysis using antiserum raised against purified recombinant PE protein (11.0.2 kDa His-fusion protein) in mice revealed a single band at 11.9 kDa with IPTG induced whole cell extract expressing recombinant PE as well as purified recombinant PE protein (Fig. 2, lanes 3, 4). No such band was visible in M15 cells as well as un-induced total cell extract of M15 harboring pQEPE plasmid (Fig. 2, lanes 1, 2). However, a band of 9.7 kDa was also detected with native protein from Map (Fig. 2, lane 5). Further the recombinant PE protein was also recognized by the serum from goat naturally infected with Map (Fig. 3).

### Deduced amino acid analysis

The nucleotide sequence of the pQEPE plasmid having 300 bp PE gene of Map has been deposited in Gene bank database under accession no. FJ716631. The predicted 99 amino acids of the 300 bp gene fragment had a mature protein of 9.7 kDa. Analysis of the deduced 99 amino acids sequence indicated that the protein contained three major hydrophobic regions (amino acids 5–10, 45–50 and 90–95).

### Induction of DTH response

**Fig. 4:**
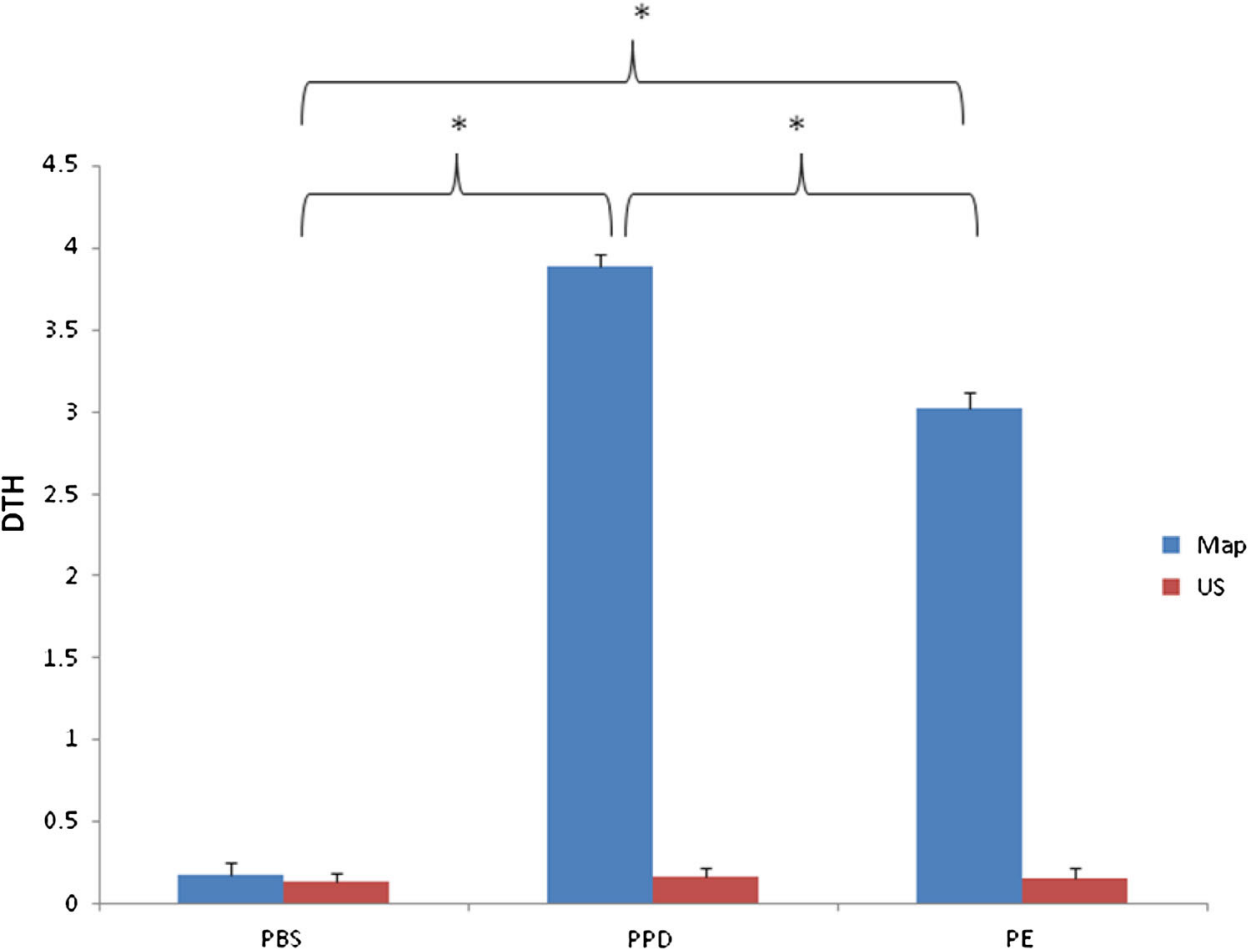
DTH (mean ± SEM) elicited by purified recombinant PE protein in *M.a. paratuberculosis* sensitized mice (mean diameter of erythema in mm) upon recall with 10 μg of the indicated antigen.*Significant difference at *P* < 0.05

Mice immunized with recombinant PE protein reacted with recombinant PE protein giving skin reaction. However, response to johnin PPD was greater than those to recombinant PE protein; non-immunized animals did not show significant skin reactions to the fusion protein as well as johnin PPD (Fig. 4).

## Discussion

PE family proteins of Mycobacterium species are known to be important antigens for conferring host immune defence. A few antigens from the PE family protein Mtb and Map have reported to elicit T cell response (Delogu and Brennan 2001) or some of them are involved to mediate humoral immune response (Narayana et al. 2007). The completion of Map genome has provided information about several ORFs from PE family proteins. In the present study, we have selected a sequence of 300 bp from the Map genome and *E. coli* expression system pQE30 UA, to produce 9.7 kDa protein of Map for further studies.

The whole cell extract of *E. coli* harboring plasmid pQE PE induced by IPTG showed a predominant band of 11.9 kDa corresponding to expected size of PE fusion protein. The expressed recombinant 6X His tag fusion protein could be purified by Ni–NTA affinity chromatography. Although a variety of heterologous expression systems have been developed to produce recombinant proteins, the purification of proteins obtained still remains problematic (Goswami et al. 1996). The presence of 6X-histidine residues at N-terminal of the fusion protein enabled us to purify the protein by single step affinity purification by using Ni–NTA resin (Deb and Goswami 2010). In the present study, recovery of the purified PE fusion protein was more than 90 % after 6 h of induction. Similarly, cloning in pQE series expression vector system was utilized to generate high levels of 35 kDa protein of Map (Basagoudanawar et al. 2005) and a 26 kDa protein of *Brucella abortus* (Kumar et al. 2008) and 34.9 kDa PPE antigen of Map (Deb and Goswami 2010). The polyclonal sera raised in mice against the recombinant His-PE protein recognized both the *E. coli* expressed PE protein as well as the PE protein in Map culture sonicate on dot and western blot analysis indicating that the immunogenic nature of PE protein was not affected by expression in a heterologous expression system.

Delay type hypersensitivity (DTH) reaction is an indicator of T cell mediated immunity towards mycobacterial infection (Orme et al. 1993). In the present study we observed the DTH responses in all the animals sensitized with Map using recombinant PE protein as well as johnin PPD. A better response was detected in the groups sensitized with Johnin PPD which may be due to polyclonal activation of T cells by multiple antigenic components in these preparations, compared to the single antigenic component of the recombinant PE protein. These results corroborated with earlier findings by Deb and Goswami (2010). This piece of information may postulate that Map PE may be a potent immune stimulating antigen and can be used as reference antigen for developing novel recombinant therapeutics/diagnostics against paratuberculosis infection.
